# Harnessing the Gut Microbes of Low Abundance With a Bent‐Capillary‐Centrifugal‐Driven (BCCD) Microdroplet Method

**DOI:** 10.1002/mbo3.70224

**Published:** 2026-02-02

**Authors:** Min‐Zhi Jiang, Zi‐Wei Zhang, Zhi Wang, Xiao‐Yang Zhu, Rashidin Abdugheni, He Jiang, Yulin Wang, Zong‐Ji Wang, Liang Zhang, Yong‐Qiang Cheng, Shuang‐Jiang Liu

**Affiliations:** ^1^ State Key Laboratory of Microbial Technology Shandong University Qingdao China; ^2^ Institute of Eco‐Environmental Forensics, School of Environmental Science and Engineering Shandong University Qingdao China; ^3^ School of Life Science Shandong University Qingdao China; ^4^ Department of Microbiology, School of Basic Medical Sciences Xinjiang Medical University Urumqi China; ^5^ Institute of Regenerative Medicine Innovation Linyi University Linyi China; ^6^ Laoshan Laboratory Qingdao China; ^7^ State Key Laboratory of Microbial Diversity and Innovative Application, and Environmental Microbiology Research Center (EMRC), Institute of Microbiology Chinese Academy of Sciences Beijing China

**Keywords:** bent‐capillary‐centrifugal‐driven (BCCD), cultivation, gut microbes at low abundance, microdroplets and encapsulating, novel taxa

## Abstract

Gut microbe cultivation is essential for studying host‐microbiota interactions. Traditional cultivation methods often fail to recover microbial species at low abundance (< 0.1%). To overcome this limitation, we employed the bent‐capillary‐centrifugal‐driven (BCCD) method to encapsulate and cultivate fecal microbes in microdroplets. Fecal bacterial cells were distributed into ~50 nL microdroplets via the BCCD generator, and the microdroplets were dispersed in the oil phase and further incubated under controlled conditions. The BCCD method significantly increased the frequency of microbes at low abundance. Compared to the plate‐based method, BCCD‐based cultivation produced distinct microbial community structures and exhibited significantly lower temporal variation during cultivation (*p* < 0.05). Lineage‐specific effect size (LEfSe) analysis revealed that BCCD‐based cultivation enriched 29 low‐abundant bacterial genera, whereas the plate‐based method enriched 26. Using this method, we isolated 1,049 bacterial strains representing 123 species and 58 genera, including 8 novel species. Among the isolated and cultivated genera, 62.1% (36/58) were microbes of low abundance in the original fecal sample, and 41.4% (12/29) of the BCCD‐specific enriched genera were successfully obtained. Notably, comparison with four major gut microbial culture studies revealed 45 species were exclusively recovered in this work. Taken together, the results demonstrated that our BCCD‐based cultivation method effectively enriched and facilitated the isolation and cultivation of microbes at low abundance and novel gut bacterial species.

## Graphical abstract

A BCCD‐based microdroplet cultivation strategy markedly enriches low‐abundance gut microbes in fecal samples, reshaping community structure and enabling efficient isolation of novel bacterial taxa. This approach provides an effective complementary tool for expanding cultivable gut microbial diversity.

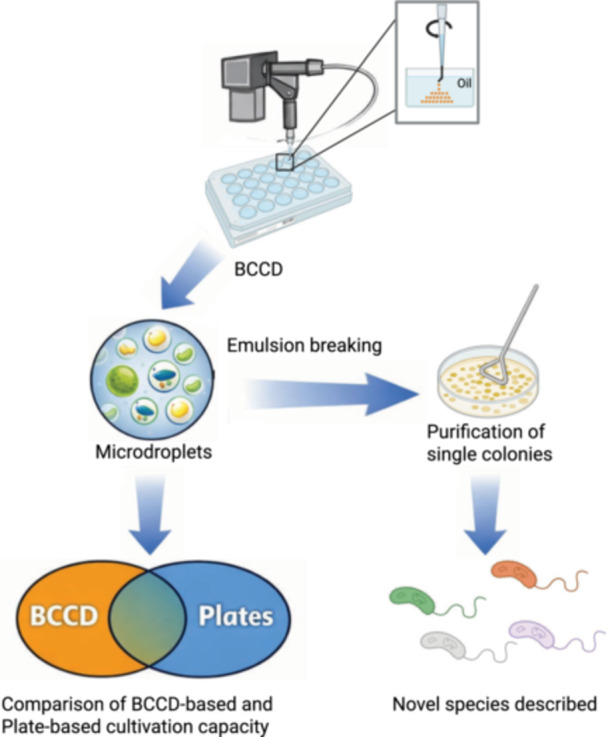

## 1 Introduction

A vast number of bacteria inhabit the human gut, and their critical roles in host health and diseases have been increasingly recognized (Fan and Pedersen 2021; Lynch and Hsiao 2019). The cultivability of gut microbes is essential for the understanding of host‐microbiota interactions (Jiang et al. 2024b; Liu et al. 2024) as well for the exploration of interactions among gut microbes (Jiang et al. 2022; Jiang et al. 2024a; Zhu et al. 2025; Xu et al. 2024). Researchers have made great efforts in the cultivation of gut microbes, for example the HBC (Forster et al. 2019), SPORE (Browne et al. 2016), hGMB (Liu et al. 2021), BIO‐ML (Poyet et al. 2019), LchspGMB (Abdugheni et al. 2022), ChrisGMB (Sun et al. 2024) and the RAGMB (Huang et al. 2024) were reported. Efforts were also made to improve the cultured microbial diversity by applying various pretreatments, including antibiotic supplementation (Yang et al. 2019; Harada et al. 2012; Ruoff et al. 1988; Tahrani et al. 2015), ethanol or heat treatments (Liu et al. 2021; Browne et al. 2016), aiming to suppress dominant taxa and to enrich stress‐tolerant ones. While these treatments enhanced cultured microbial diversity to some extent, they did not overcome the difficulty to recover gut microbes at low abundance.

To overcome the limitations on gut microbial cultivability, alternative approaches have been explored. Microfluidic platforms have emerged as powerful tools for high‐throughput microbial isolation. Typically, cells are loaded into microdroplets based on a Poisson distribution (Villa et al. 2020). In practice, this method generates a large number of empty microdroplets to avoid that more than one cell being dispensed in one microdroplet, which costs more chemicals, labors, and complicates downstream experiments. Recently, a method has been developed that allows the detection of a single cell within microdroplets, filtering each droplet containing one cell into a culture tube (Diao et al. 2022). These systems often impose strict physical isolation, which can impair the growth of microbes that depend on cross‐feeding or quorum sensing (Lewis et al. 2021). Another method integrates image‐based colony recognition and machine learning to select unique colonies based on morphological features (Huang et al. 2023). Still, the recruitments of gut microbes at low abundance and microbial interactions are challenging.

In this study, we addressed those challenges by implementing a bent‐capillary‐centrifugal‐driven (BCCD) droplet pre‐cultivation strategy. This system utilizes a BCCD droplet generator (Zhang et al. 2023), to disperse fecal suspensions into thousands of uniform microdroplets. Each droplet contains a random mixture of microbes, avoiding excessive competition from dominant taxa. After preculturing for 5 days, we observed that the sum of low‐abundant taxa (relative abundance < 0.1%) in the fecal sample increased from 9.8% to 78.3%. Using this approach, we successfully isolated 1,049 colonies, representing 123 species, including 8 potential novel species. Importantly, 45 species were exclusively isolated in this study compared to previous large‐scale efforts on GMBs, highlighting the unique advantage of this method for capturing previously uncultured or underrepresented taxa. We anticipate that this method will serve as a powerful addition to the gut microbial cultivation toolbox and facilitate the discovery of low‐abundant gut microbes.

## 2 Materials and Methods

### 2.1 Sample Collection and Pretreatment

All fecal samples used in this experiment were collected from the same volunteer, a healthy 20‐year‐old male who had not taken antibiotics within the past 3 months. Samples were collected and transferred freshly (not frozen) to an anaerobic workstation with a gas composition of 85 N_2_%, 5 CO_2_%, and 10 H_2_%. Fecal samples were diluted and filtered as described in our previous study (Jiang et al. 2024a). The filtrates were further diluted with Modified Gifu Anaerobic Medium (mGAM), Yeast Extract‐Casein Fatty Acid Medium‌ (YCFA), or m104b medium (for composition of each medium, see Table S1). For ethanol‐treated samples, 1 mL of filtrate was centrifuged at 6,000 rpm for 10 min at room temperature, and the supernatant was discarded. Then 30%, 50%, or 70% ethanol was added to 1 mL for incubation times of 5, 30, or 60 min. The ethanol‐treated samples were then centrifuged again, the supernatant was discarded, and the pellet was washed twice with 1 mL PBS before resuspending in 1 mL of mGAM medium containing 6.9 mmol/L sodium taurocholate and 10 mM mixed amino acid (Wheeldon et al. 2011). For antibiotics treatment, the dilutions were prepared in mGAM, YCFA, or m104b medium supplemented with 5 or 30 mg/L of ampicillin or vancomycin (Yang et al. 2019; Harada et al. 2012; Ruoff et al. 1988; Tahrani et al. 2015).

### 2.2 Microdroplet Generation and Culture Conditions

The BCCD droplet generator used in this study was adapted from a previously described system (Zhang et al. 2023). Briefly, the BCCD device consisted of a portable rotary motor (Shanghai Hengchuan Machinery Equipment Co. Ltd., China), an integrated device of a bent capillary and pipette (IDBCP), and a flat‐bottom 48‐well plate. For droplet generation, the oil phase consisted of n‐tetradecane (Macklin, China) supplemented with 2% (v/v) EM90 surfactant (Evonik, Germany). The oil phase was sterilized by filtration through a 0.22 μm membrane filter and added to the wells of a 48‐well plate (750 μL per well). Fecal samples were homogenized and diluted with the appropriate culture medium (mGAM, YCFA, or m104b). Subsequently, 25 μL of fecal sample dilution was accurately loaded into the bent‐capillary. During droplet generation, the rotary motor was first accelerated and then maintained at a constant rotational speed. The bent‐capillary tip was immersed into the oil phase only after reaching this constant speed, enabling the reproducible formation of monodisperse water‐in‐oil (W/O) microdroplets. Under these conditions, the fecal sample was dispersed into uniform microdroplets with an approximate volume of 50 nL, resulting in the random partitioning of microbial cells among droplets. The generated microdroplets were maintained dispersed in the oil phase within the 48‐well plate and incubated statically at 37°C for 5 or 10 days under anaerobic conditions. The plate‐based cultivation groups were prepared by spreading 25 μL of the same fecal sample homogenate onto the surface of agar media and incubating under identical conditions. After incubation, five samples were collected from each culture condition to serve as biological replicates. Cells were then harvested either by centrifugation for emulsion breaking or by scraping the agar surface to ensure complete recovery from all samples.

### 2.3 Cell Collection, Genomic Extraction, and Sequencing

After incubation, microdroplets were centrifuged at 6,000 rpm for 10 min to remove the oil phase. For agar medium samples, colonies were scraped from the plates. DNA was extracted using the Qiagen PowerSoil Kit (Qiagen, Germany) according to the manufacturer's instructions. To assess the impact of the 2 culture methods and different incubation times on the community, purified DNA was subjected to amplicon sequencing to obtain community profiles using the Illumina NovaSeq PE250 platform, targeting the V3‐V4 regions. Data assembly (Demux) and filtering (DADA2) were performed using QIIME2 (v 23.5) (Bolyen et al. 2019). Representative sequences were obtained using feature‐table summarize, and taxonomic classification was performed with feature‐classifier classify‐sklearn. Following the creation of the feature table, downstream analyses were conducted using amplicon sequence variants (ASVs) annotated with the SILVA database (Quast et al. 2013). Principal coordinate analysis (PCoA) was visualized using the R package vegan (v2.6‐10). Differences in genera or ASVs were analyzed using LEfSe (Chen et al. 2022).

### 2.4 Bacterial Isolation and Cultivation

Fecal samples for isolating gut microbes were obtained from the same donor, who provided written informed consent. The collected samples were prepared into microdroplets as previously described and incubated at 37°C to harvest cells. These cells were then plated onto mGAM, YCFA, or m104b agar medium, and single colonies were picked for transfer to the same liquid medium. To identify the isolated species, we amplified the 16S rRNA gene using primers 27 F (AGAGTTTGATCMTGGCTCAG) and 1492 R (GGTTACCTTGTTACGACTT), following the PCR methods described in previous work (Jiang et al. 2022). The PCR products were subsequently sequenced using Sanger sequencing, with taxonomic classification and annotation information determined through BLAST (https://blast.ncbi.nlm.nih.gov/Blast.cgi) and EzBioCloud (https://www.ezbiocloud.net/).

### 2.5 Polyphasic Characterization and Nomenclature of Novel Taxa

The identification of potential novel taxa was based on the analysis of each type of strain in terms of phylogenetic, genomic, and morphological characteristics, as described in previous work (Liu et al. 2021). For each potential novel species, a phylogenetic tree was constructed using the 16S rRNA gene sequences of the strains from phylogenetically related genera and species. The tree was generated with MEGA11 using the neighbor‐joining method to illustrate the phylogenetic distribution and taxonomic relationships of each potential novel taxon and its closely related taxa. Additionally, a genome‐based phylogenomic tree for each potential novel species was constructed using Composition Vector Tree Version 3 (http://cvtree.net/v3/cvtree/index.html). The closely related taxa on both the phylogenetic and phylogenomic trees were used for further genome‐based analysis. This analysis included calculating the average nucleotide identity (ANI) and digital DNA‐DNA hybridization (dDDH). ANI values and heatmaps were generated using the OrthoANI OTA software (Lee et al. 2016), while the dDDH values between the draft genome of the potential novel species and its phylogenetically and phylogenomically closest genomes were calculated using the Genome‐to‐Genome Distance Calculator 3.0 (GGDC) (Meier‐Kolthoff et al. 2022). Bacterial cell morphology was observed using a transmission electron microscope (TEM), Tecnai G2 F20 (FEI, USA). A taxon was considered a potential novel species if it met all of the following three criteria: (1) 16S rRNA sequence identity < 98.7%, (2) dDDH value < 70%, (3) ANI < 95%. If the ANI value is > 95% but the dDDH value is < 70%, the strain is still considered a novel species. Genome sequencing and analysis were performed by Kindstar Sequenon Biotechnology (Wuhan) Co. Ltd.

### 2.6 Data Processing and Figures

All data are presented as means ± SD. Data analysis and figure modifications were conducted using GraphPad Prism 9.0 software, BioRender (https://biorender.com), and Adobe Illustrator 2019. Statistical significance was evaluated using pairwise Student's *t*‐tests, and *p* values were categorized as follows: *p < 0.05, **p < 0.01, ***p < 0.001.

## 3 Results

### 3.1 Microdroplets Encapsulation and BCCD‐Based Cultivation of Gut Microbiota

**Figure 1:**
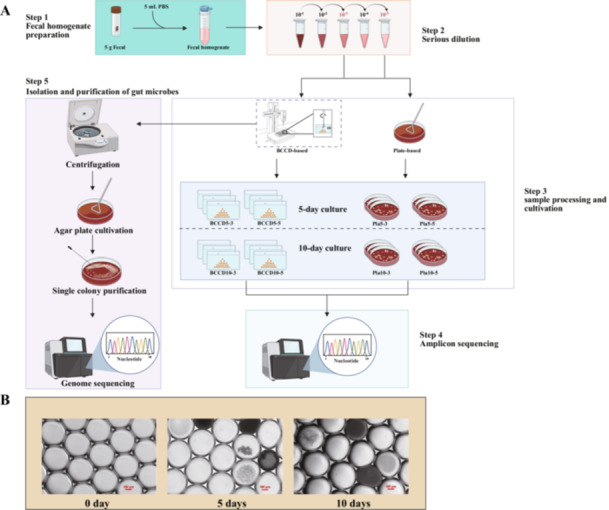
The experimental design, encapsulation of fecal samples, and cultivation of microdroplets. (A) The fecal bacterial cell encapsulation and cultivation. The fecal samples were diluted with mGAM medium, and each of the 25 μL of the 10^−3^ and 10^−5^ dilutions were spread onto the mGAM agar plate (plate‐based cultivation method, called Pla group), or encapsulated into microdroplets using BCCD generator and dispensed into an oil phase (BCCD‐based cultivation method, called BCCD group). In this diagram, all cultivations were labeled, for example, a cultivation from the BCCD‐based experiments at 5 days and a dilution of 10^−3^ was labeled as “BCCD 5–3”. (B) Representative pictures of microdroplets and bacterial growth in the microdroplets at 0, 5, and 10 days of cultivation.

We employed a BCCD microdroplets generator (Zhang et al. 2023) for the encapsulation of fecal bacterial cells. After dilution, fecal bacterial cells were serially dispersed into ~50 nL microdroplets and randomly dispensed into 48‐well plates that contained 750 μL oil. This process ensured the random distribution of fecal bacterial cells into individual droplets for subsequent incubation. For comparison, we worked on the same fecal sample with traditional plate‐based cultivation in parallel. The experimental workflow is illustrated in Figure 1A. Samples were cultivated with mGAM medium for downstream experiments. Following quality control and denoising, we obtained 3.3 Gbp of 16S rRNA sequence data. A total of 3,540 ASVs were annotated (Table S2).

### 3.2 BCCD Droplet Method Enriched Low‐ Abundant Microbial Species

**Figure 2:**
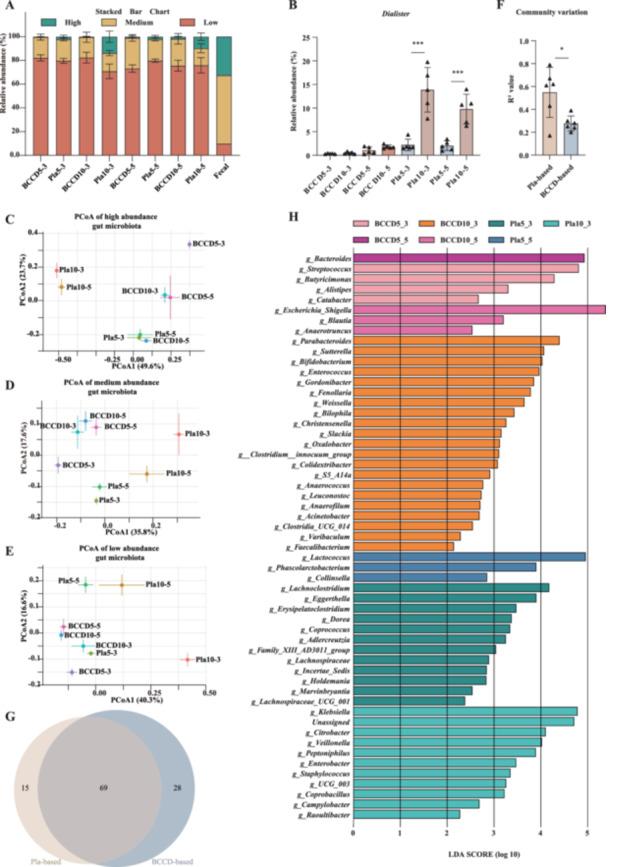
The composition of microbial communities in BCCD‐based cultivation and plate‐based cultivation methods. (A) Stacked bar chart showing the proportions of high, medium, and low abundance taxa in the samples. (B) The relative abundance variation of *Dialister*. (C–E) The PCoA analysis of microbial communities. (F) Comparison of R^2^ values between the BCCD‐based and plate‐based methods. (G) Venn diagram at the genus level comparing taxa cultivated by the BCCD‐based and plate‐based methods. (H) Gut microbiota bacterial comparisons between BCCD‐based and Pla‐based cultivation methods were analyzed by LEfSe (LDA > 2, *p* < 0.05). LDA scores were calculated by LDA effect size, using linear discriminant analysis to assess the effect size of each differentially abundant bacterial taxa. Statistical significance was evaluated using pairwise Student's *t*‐tests, and *p* values were categorized as follows: *p < 0.05, **p < 0.01, ***p < 0.001.

To systematically assess the enrichment capability of the BCCD‐based cultivation method in comparison with the traditional plate‐based method, fecal microbial species were categorized into three abundance levels: low (< 0.1%), medium (0.1%–1%), and high (> 1%). Both cultivation methods substantially enriched low abundance gut microbes in the fecal sample, increasing their combined relative abundance from 9.8% to 78.3% with the BCCD‐based method and to 76.5% with the plate‐based method (Figure 2A). As the proportion of low abundance taxa increased, a corresponding decrease was observed in medium‐abundance taxa (from 57.7% to 16.4% and 20.9%, respectively) and high abundance taxa (from 32.5% to 0.9% and 7.0%, respectively). We observed that the relative abundance of *Dialister*, a high‐abundance microbe in the fecal sample, significantly increased in the Pla‐based culture method as the incubation time increased, but not in the BCCD‐based method (Figure 2B). To evaluate whether the cultured microbial communities from the BCCD‐based and plate‐based methods, we conducted a principal coordinate analysis (PCoA). The results revealed that the community compositions at the same time points significantly diverged between the BCCD‐based and plate‐based methods (pairwise PERMANOVA, *p* < 0.05; Figure 2C–E, Table S3). Temporal variations in community structure were observed in microbial communities that were obtained with both methods. We observed that the variation of microbial communities over time was less in the BCCD‐cultivated communities compared to that in the plate‐based method (Figure 2F). We further assessed the cultivation capacities of the two methods for different microbial taxa using a Venn diagram. The results showed that a total of 112 genera could be cultivated by the two methods combined, of which 61.6% (69/112) were shared between both approaches. In contrast, the BCCD‐based and plate‐based methods uniquely cultivated 25.0% (28/112) and 13.4% (15/112) of the genera, respectively (Figure 2G). This suggests that the BCCD‐based method would provide more opportunity to extract low‐abundant microbes from fecal samples.

Both the BCCD‐based and plate‐based cultivation methods effectively increased the proportion of low‐abundant microbes in the fecal sample. To further assess and compare their enrichment capabilities for these taxa, we conducted a linear discriminant analysis effect size (LEfSe) analysis at the genus level. Genera with LEfSe scores > 2 were considered significantly enriched (Figure 2H). The LEfSe results revealed that BCCD‐based cultivation enriched 29 genera, including several gut commensals with known or potential probiotic properties, such as *Bifidobacterium* (Liu et al. 2017), *Christensenella* (Liu et al. 2024), *Parabacteroides* (Wang et al. 2019; Qiao et al. 2022), and *Blautia* (Hosomi et al. 2022; Ye et al. 2023). In contrast, plate‐based cultivation enriched 26 genera, notably including members of the *Dorea* (Dahl et al. 2020; Mi et al. 2023; Abdugheni et al. 2022), *Coprococcus* (Notting et al. 2023), which are also important microbes in the human gut. In summary, the BCCD‐based cultivation method exhibits a unique enrichment profile, making it a promising complementary strategy to conventional plate‐based approaches for the isolation and cultivation of gut microbes.

### 3.3 The BCCD Method Is Effective for Harvesting Diverse Gut Microbes

**Figure 3:**
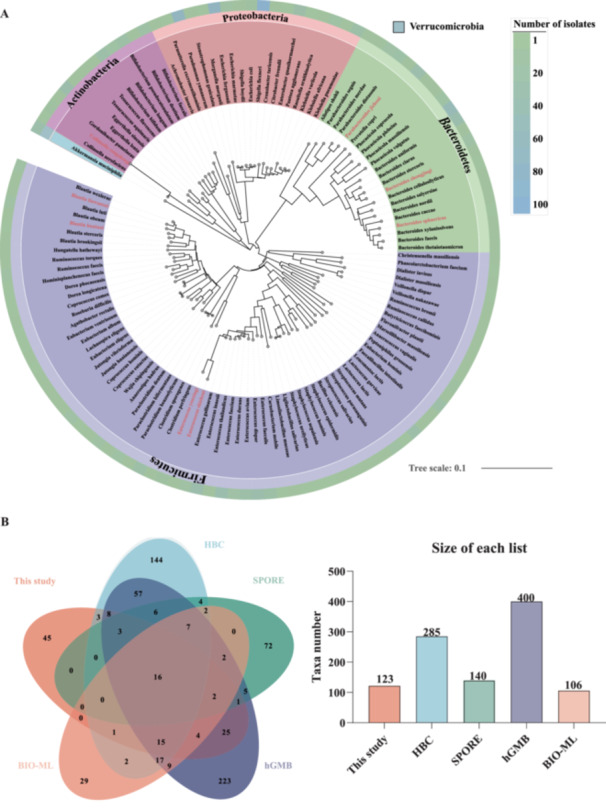
Composition and characteristics of isolated microbes from the human gut. (A) The phylogenetic tree was constructed with the neighbor‐joining method based on full‐length 16S rRNA genes of 123 representative strains and visualized with iTOL (Letunic and Bork 2021). The label backgrounds are color coded by the 5 phyla, while the outer ring heatmap displays the isolate counts. Species highlighted in red correspond to novel taxa proposed in this study. (B) Comparative analysis of gut bacterial diversity with HBC (Forster et al. 2019), SPORE (Browne et al. 2016), hGMB (Liu et al. 2021), BIO‐ML (Poyet et al. 2019). Venn diagram showing the five large‐scale cultivation studies and overlapping numbers at the species level. (Forster et al. 2019), SPORE (Browne et al. 2016), hGMB (Liu et al. 2021), BIO‐ML (Poyet et al. 2019). Venn diagram showing the five large‐scale cultivation studies and overlapping numbers at species level.

To experimentally evaluate the effectiveness of the BCCD‐based method in isolating low‐abundant microbes, we collected fecal samples and applied BCCD‐based cultivation conditions for bacterial isolation. We implemented 14 different pretreatments (Table S4) that were shown to be effective (Abdugheni et al. 2022; Liu et al. 2021; Ruoff et al. 1988; Yang et al. 2019) for BCCD‐based enrichment. Following the enrichment, cultures from the 48‐well plates were spread on conventional agar plates and single colonies formed on the surface of the agar were picked. In total, we obtained 1,049 bacterial strains and their full‐length 16S rRNA genes were sequenced and aligned using BLAST and EzBioCloud to determine their taxonomic identity. Using a 98.7% sequence similarity threshold for species clustering, the 1,049 strains were phylogenetically classified into 123 species, representing 58 genera (Figure 3A, Table S5). Representative genera significantly enriched in Figure 2H, such as *Bacteroides*, *Bifidobacterium*, *Christensenella*, *Enterococcus*, and *Parabacteroides*, were successfully isolated. Among the isolated genera, 62.1% (36/58) corresponded to low‐abundant taxa in the original fecal sample, and 41.4% (12/29) of the BCCD‐specific enriched genera identified by LEfSe analysis were successfully isolated. Based on the isolation results, the BCCD‐based cultivation method effectively enriched low‐abundant genera and improved the isolation efficiency. We compared the BCCD cultivation with previous large‐scale gut microbial collections, including HBC (Forster et al. 2019), SPORE (Browne et al. 2016), hGMB (Liu et al. 2021) and BIO‐ML (Poyet et al. 2019). These five large‐scale culture collections were established in recent years through extensive isolation and cultivation of strains from healthy individuals, primarily relying on traditional plate‐based purification of single colonies. Comparison with these collections highlights the diversity of strains recovered in our study and demonstrates that the BCCD‐based method enables the recovery of species that are difficult to obtain using conventional cultivation approaches. The results demonstrated that BCCD‐based cultivation successfully covered 45 unique species that were not covered in the previously large‐scale cultivations (Figure 3B).

Bacterial strains that showed 16S rRNA sequence similarities below 98.7% to any previously reported bacterial species were subjected to genome sequencing. Results showed that 8 strains represented previously uncultured gut microbes at the species level. The eight potentially novel species were characterized and their taxonomies were determined with the polyphasic method (see the next section).

### 3.4 Characterization of Novel Species From the BCCD Method

**Figure 4:**
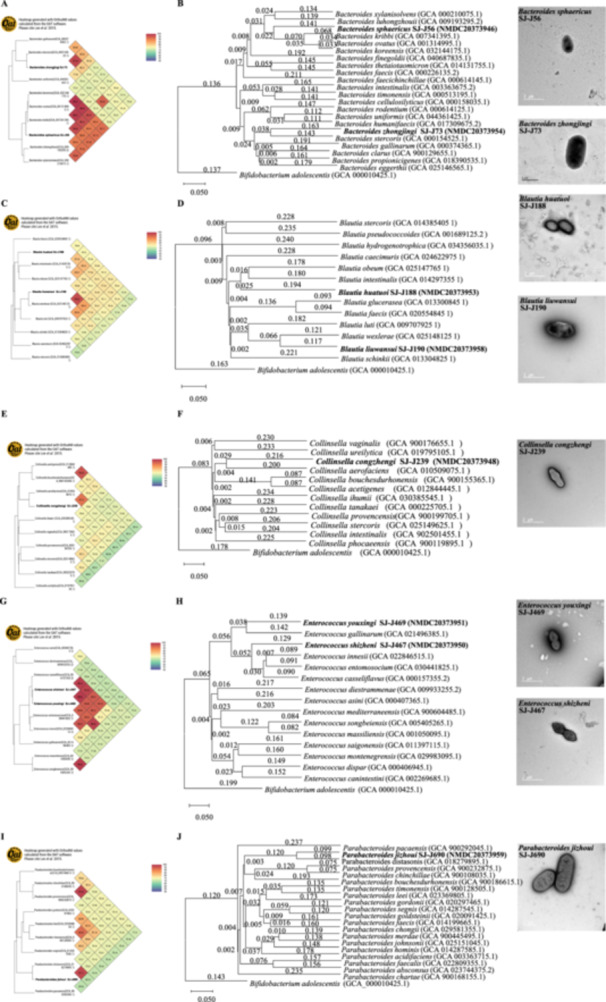
Phylogenetic relationships and TEM morphology of 8 novel species. (A, C, E, G, I) OrthoANI heatmaps of 8 representative strains. (B, D, F, H, J) Phylogenomic trees generated using an alignment‐free, whole‐genome approach (CVTree3) (Zuo and Hao 2015), alongside TEM images of cell morphology. Refseq IDs of representative genomes are given in parentheses.

The 8 potentially novel species were genome sequenced, their phenotypic characteristics were determined, and are summarized in Table 1, with additional details available in the “Supplementary Taxon Data”. These species belonged to five genera (Figure 4) and exhibited morphological diversity, including rod‐shaped, blunt‐ended, oval, and spherical forms. Genome statistics for these species are presented in Table S6. Taxonomic characterization and proposals were based on a combination of phylogenetic analyses, morphological observations, and genome‐based assessments. All species were named in accordance with the rules of the International Code of Nomenclature of Prokaryotes (ICNP), and their protologs are provided in Table 1. All genome sequences have been made publicly available through the China National Microbiology Data Center (NMDC). Moreover, all 8 novel strains were isolated from the faeces of a healthy adult and are deposited in China General Microorganisms Culture Center (CGMCC).

**Table 1:** The protologs of 8 novel species.

Types	Names	Rank	Etymology	Description, type designation, and accession number
Strain SJ‐J56^T^	*Bacteroides sphaericus*	sp. nov.	sphae'ri. cus. L. masc. adj. *sphaericus*, spherical, referring to the spherical cell morphology	OrthoANI heatmap of strain SJ‐J56^T^ is shown in Figure 4A, and the genome‐based phylogenomic tree is presented in Figure 4B. Cells are strictly anaerobic, spherical with a single blunt end. Growth to stable phase occurs after 72 h incubation in 104b medium at 37°C, pH = 7.0–7.5. The genomic DNA G + C content is 42.7 mol%. The type strain SJ‐J56^T^ is deposited as CGMCC 1.58709, with the NMDC accession number is NMDC20373946.
Strain SJ‐J73^T^	*Bacteroides zhongjingi*	sp. nov.	zhong. jing'i. N.L. gen. masc. n. *zhongjingii*, named after Zhongjing Zhang, the Eastern Han dynasty physician revered as the “Sage of Medicine”.	OrthoANI heatmap of strain SJ‐J73^T^ is shown in Figure 4A, and the genome‐based phylogenomic tree is presented in Figure 4B. Cells are strictly anaerobic, oval, or spherical. Growth to stable phase occurs after 72 h incubation in 104b medium at 37°C, pH = 7.0–7.5. The genomic DNA G + C content is 43.7 mol%. The type strain SJ‐J73^T^ is deposited as CGMCC 1.58710, with the NMDC accession number is NMDC20373954.
Strain SJ‐J188^T^	*Blautia huatuoi*	sp. nov.	hua. tuo'i. N.L. gen. masc. n. *huatuoi*, in honor of Tuo Hua, the pioneer of ancient Chinese surgical techniques and anesthesia.	OrthoANI heatmap of strain SJ‐J188^T^ is shown in Figure 4C, and the genome‐based phylogenomic tree is presented in Figure 4D. Cells are strictly anaerobic, spherical or oval, or short rod. Growth to stable phase occurs after 72 h incubation in 104b medium at 37°C, pH = 7.0–7.5. The genomic DNA G + C content is 43.7 mol%. The type strain SJ‐J188^T^ is deposited as CGMCC 1.58711, with the NMDC accession number is NMDC20373953.
Strain SJ‐J190^T^	*Blautia liuwansui*	sp. nov.	liu. wan. su'i. N.L. gen. masc. n. *liuwansui*, named after the Chinese medical scientist Wansu Liu.	OrthoANI heatmap of strain SJ‐J190^T^ is shown in Figure 4C, and the genome‐based phylogenomic tree is presented in Figure 4D. Cells are strictly anaerobic, oval or short rod shaped. Growth to stable phase occurs after 72 h incubation in 104b medium at 37°C, pH = 7.0–7.5. The genomic DNA G + C content is 41.2 mol%. The type strain SJ‐J190^T^ is deposited as CGMCC 1.58713, with the NMDC accession number is NMDC20373958.
Strain SJ‐J239^T^	*Collinsella congzhengi*	sp. nov.	cong. zheng'i. N.L. gen. masc. n. *congzhengi*, named in honor of Congzheng Zhang, a prominent Chinese physician of the Jin‐Yuan dynasty, recognized for his pioneering work in purgative therapy and the theory of pathogenic factors in traditional Chinese medicine.	OrthoANI heatmap of strain SJ‐J239^T^ is shown in Figure 4E, and the genome‐based phylogenomic tree is presented in Figure 4F. Cells are strictly anaerobic, club shaped rods with a single blunt end. Growth to stable phase occurs after 72 h incubation in 104b medium at 37°C, pH = 7.0–7.5. The genomic DNA G + C content of the type strain is 47.9 mol%. The type strain SJ‐J239^T^ is deposited as CGMCC 1.58714, with the NMDC accession number is NMDC20373948.
Strain SJ‐J467^T^	*Enterococcus shizheni*	sp. nov.	shi. zhen'i. N.L. gen. masc. n. *shizheni*, named in honor of Shizhen Li, whose encyclopedic Compendium of Materia Medica revolutionized pharmacognosy.	OrthoANI heatmap of strain SJ‐J467^T^ is shown in Figure 4G, and the genome‐based phylogenomic tree is presented in Figure 4H. Cells are strictly anaerobic, ovoid in shape, elongated along the chain direction, and appear in pairs. Growth to stable phase occurs after 72 h incubation in 104b medium at 37°C, pH = 7.0–7.5. The genomic DNA G + C content is 50.4 mol%. The type strain SJ‐J467^T^ is deposited as CGMCC 1.58715, with the NMDC accession number is NMDC20373950.
Strain SJ‐J469^T^	*Enterococcus youxingi*	sp. nov.	you. xing'i. N.L. gen. masc. n. *youxingi*, named in honor of Youxing Wu, who pioneered the concept of epidemic pathogens as distinct entities in traditional Chinese medicine.	OrthoANI heatmap of strain SJ‐J469^T^ is shown in Figure 4G, and the genome‐based phylogenomic tree is presented in Figure 4H. Cells are strictly anaerobic, ovoid in shape, elongated along the chain direction, and appear in pairs. Growth to stable phase occurs after 72 h incubation in 104b medium at 37°C, pH = 7.0–7.5. The genomic DNA G + C content is 41.1 mol%. The type strain SJ‐J469^T^ is deposited as CGMCC 1.58716, with the NMDC accession number is NMDC20373951.
Strain SJ‐J690^T^	*Parabacteroides jizhoui*	sp. nov.	ji. zhou'i; N.L. gen. masc. n. *jizhoui*, named in honor of Jizhou Yang, whose codified classical acupuncture theories.	OrthoANI heatmap of strain SJ‐J690^T^ is shown in Figure 4I, and the genome‐based phylogenomic tree is presented in Figure 4J. Cells are strictly anaerobic, oval, spherical with a single blunt end. Growth to stable phase occurs after 72 h incubation in 104b medium at 37°C, pH = 7.0–7.5. The genomic DNA G + C content is 45.03 mol%. The type strain SJ‐J690^T^ is deposited as CGMCC 1.58721, with the NMDC accession number is NMDC20373959.

## 4 Discussion

Recent studies have emphasized the important functional roles of low‐abundant members of the gut microbiota in modulating host physiology (Han et al. 2022; Han and Vaishnava 2023; Cena et al. 2021). Traditional plate‐based cultivation effectively obtained the common gut microbes (Liu et al. 2021; Browne et al. 2016). The primary goal of this study was to isolate and cultivate low‐abundant microbes from fecal samples. With the application of the BCCD‐based cultivation, we successfully isolated and cultivated low‐abundant species of *Christensenella*, *Enterococcus*, and *Parabacteroides* from fecal samples. The BCCD‐based method adopted encapsulation and microdroplets techniques, and has advantages over the traditional plate‐based cultivation. 1) Encapsulation disperses nutrients and cells into individual microdroplets, where cell growth occurs in a spatially confined environment. This physical separation restricts dominant or fast‐growing cells to their own microdroplets, thereby providing low abundance or slow‐growing cells with the opportunity to proliferate without competition. 2) Single‐cell approaches such as microfluidics have the advantages of cell sorting and isolation (Meng et al. 2022; Diao et al. 2022). Instead, the BCCD‐based method randomly distributes and encapsulates multiple and heterogeneous microbial cells within microdroplets. This would allow cellular interactions and would provide the possibility to promote the cultivation of microbes that rely on each other for growth, which might be partly the reason that we obtained highly diverse microbial taxa from BCCD‐based than from traditional agar plate methods. In the future, the BCCD‐based method might be applied for cultivation of auxotroph bacteria such as the UCG group, a group of commensal gut bacteria that has not been previously cultivated and is strongly associated with human health (Feng et al. 2022; Warmbrunn et al. 2024; Wang et al. 2023).

There are limitations of the BCCD‐based cultivation method. Firstly, this is not a method that specifically targets low‐abundant microbes. The dominant microbial cells from samples occupy the majority of the microdroplets and the random encapsulation of microbial cells still poses challenges in enriching extremely low‐abundant microbial cells. Secondly, we compared the community compositions obtained from the BCCD‐based cultivation and from the traditional plate‐based cultivation. The results indicated that there were overlapping but each method enriched different microbial taxa. These findings suggest that the BCCD‐based cultivation method is not intended to fully replace traditional or other high throughput microbial cultivation methods. Thirdly, it is still necessary to apply diverse medium formulations and multiple pretreatment methods to meet the metabolic needs of more fastidious microbes, which lead to redundancy of dominant microbial taxa in samples and results in tedious work (Tramontano et al. 2018; Oberhardt et al. 2015). Future work may benefit from the integration of metabolic modeling and droplet‐based co‐culture prediction algorithms.

## 5 Conclusion

In conclusion, we have developed a BCCD‐based microdroplet cultivation method that significantly enriches low‐abundant gut microbes from fecal samples. This method is effective in enriching and culturing those otherwise difficult to be cultured, low‐abundant microbes. Collectively, our findings suggest that the BCCD‐based microdroplet cultivation offers a promising and alternative cultivation techniques and facilitates the discovery of yet‐to‐be cultivated gut microbial resources.

## Author Contributions

**Min‐Zhi Jiang:** writing – review and editing, writing – original draft, visualization, methodology, formal analysis, investigation. **Zi‐Wei Zhang:** writing – review and editing, resources, investigation, validation. **Zhi Wang:** writing – review and editing, resources, investigation. **Xiao‐Yang Zhu:** writing – review and editing, data curation, investigation. **Rashidin Abdugheni:** writing – review and editing, data curation, visualization. **He Jiang:** writing – review and editing, supervision. **Yulin Wang:** writing – review and editing, methodology. **Zong‐Ji Wang:** writing – review and editing, supervision. **Liang Zhang:** data curation, visualization. **Yong‐Qiang Cheng:** writing – review and editing, supervision, conceptualization. **Shuang‐Jiang Liu:** writing – review and editing, supervision, methodology, conceptualization.

## Conflicts of Interest

The authors declare no conflicts of interest.

## Supporting information


**Fig. S1:** Neighbour‐joining phylogenetic tree based on 16S rRNA gene sequences of strains SJ‐J56T and SJ‐J73T, together with type species of the same genus retrieved from the List of Prokaryotic names with Standing in Nomenclature. Bootstrap values were calculated from 1,000 replicates. **Fig. S2:** Neighbour‐joining phylogenetic tree based on 16S rRNA gene sequences of strains SJ‐J188T and SJ‐J190T, together with type species of the same genus retrieved from the List of Prokaryotic names with Standing in Nomenclature. Bootstrap values were calculated from 1,000 replicates. **Fig. S3:** Neighbour‐joining phylogenetic tree based on 16S rRNA gene sequences of strains SJ‐J239T, together with type species of the same genus retrieved from the List of Prokaryotic names with Standing in Nomenclature. Bootstrap values were calculated from 1,000 replicates. **Fig. S4:** Neighbour‐joining phylogenetic tree based on 16S rRNA gene sequences of strains SJ‐J467T and SJ‐J469T, together with type species of the same genus retrieved from the List of Prokaryotic names with Standing in Nomenclature. Bootstrap values were calculated from 1,000 replicates. **Fig. S5:** Neighbour‐joining phylogenetic tree based on 16S rRNA gene sequences of strains SJ‐J690T, together with type species of the same genus retrieved from the List of Prokaryotic names with Standing in Nomenclature. Bootstrap values were calculated from 1,000 replicates.

**Table S1:** The media used for gut microbe isolation. **Table S2:** The relative abundance of all ASVs and their taxonomic assignments. **Table S3:** PCoA‐based analysis of intergroup differences in microbial community composition. **Table S4:** The pretreatment methods used for gut microbe isolation. **Table S5:** The taxonomic and 16S rRNA gene information of isolated strains in this study. **Table S6:** Genome Information on 8 novel species.

## Data Availability

Data is deposited in the National Microbiology Data Center (NMDC) with the accession number of NMDC10019511 (https://nmdc.cn/resource/genomics/project/detail/NMDC10019511).
