# Supplementary material for: Harnessing the Gut Microbes of Low Abundance With a Bent‐Capillary‐Centrifugal‐Driven (BCCD) Microdroplet Method
Source: Microbiologyopen. 2026 Feb 2;15(1):e70224. doi: 10.1002/mbo3.70224 (PMC12864543; doi:10.1002/mbo3.70224)
Supplement: Supplementary file 1 — Fig. S1: Neighbour‐joining phylogenetic tree based on 16S rRNA gene sequences of strains SJ‐J56T and SJ‐J73T, together with type species of the same genus retrieved from the List of Prokaryotic names with Standing in Nomenclature. Bootstrap values were calculated from 1,000 replicates. Fig. S2: Neighbour‐joining phylogenetic tree based on 16S rRNA gene sequences of strains SJ‐J188T and SJ‐J190T, together with type species of the same genus retrieved from the List of Prokaryotic names with Standing in Nomenclature. Bootstrap values were calculated from 1,000 replicates. Fig. S3: Neighbour‐joining phylogenetic tree based on 16S rRNA gene sequences of strains SJ‐J239T, together with type species of the same genus retrieved from the List of Prokaryotic names with Standing in Nomenclature. Bootstrap values were calculated from 1,000 replicates. Fig. S4: Neighbour‐joining phylogenetic tree based on 16S rRNA gene sequences of strains SJ‐J467T and SJ‐J469T, together with type species of the same genus retrieved from the List of Prokaryotic names with Standing in Nomenclature. Bootstrap values were calculated from 1,000 replicates. Fig. S5: Neighbour‐joining phylogenetic tree based on 16S rRNA gene sequences of strains SJ‐J690T, together with type species of the same genus retrieved from the List of Prokaryotic names with Standing in Nomenclature. Bootstrap values were calculated from 1,000 replicates. [file MBO3-15-e70224-s002.docx]

Harnessing the gut microbes of low abundance with a bent-capillary-centrifugal-driven (BCCD) microdroplet method

Min-Zhi Jiang^a^, Zi-Wei Zhang^b^, Zhi Wang^c^, Xiao-Yang Zhu^a^, Rashidin Abdugheni^d^, He Jiang^a^, Yulin Wang^a^, Zong-Ji Wang^e^, Liang Zhang^a, e^, Yong-Qiang Cheng^b, f *^, Shuang-Jiang Liu^a, g *^

^a^ State Key Laboratory of Microbial Technology, Shandong University, Qingdao 266000, P. R. China.

^b^ Institute of Eco-Environmental Forensics, School of Environmental Science and Engineering, Shandong University, Qingdao 266000, P. R. China.

^c^ School of Life Science, Shandong University, Qingdao 266000, P. R. China.

^d^ Department of Microbiology, School of Basic Medical Sciences, Xinjiang Medical University, Urumqi, 830000, China.

^e^ Institute of Regenerative Medicine Innovation, Linyi University, Linyi 276000, P. R. China.

^f^ Laoshan Laboratory, Qingdao 266000, Shandong, China.

^g^ State Key Laboratory of Microbial Diversity and Innovative Application, and Environmental Microbiology Research Center (EMRC), Institute of Microbiology, Chinese Academy of Sciences, Beijing 100101, China.

*Corresponding authors: Yong-Qiang Cheng ([chengyongqiang@sdu.edu.cn](mailto:chengyongqiang@sdu.edu.cn)) and Shuang-Jiang Liu ([liusj@sdu.edu.cn](mailto:liusj@sdu.edu.cn)).

**Taxon 1:** ***Bacteroides sphaericus* sp. nov.**

The taxon 1, represented by strain SJ-J56 ^T^ is phylogenetically closest to *Bacteroides koreensis*, with 16S rRNA gene identity of 95.8 %. Phylogenetic tree shows that strain SJ-J56 ^T^ clusters with other members of the genus *Bacteroides* (Fig. S1), suggesting strain SJ-J56 ^T^ is a member of the genus *Bacteroides*. The genome of strain SJ-J56 ^T^ was sequenced and the NMDC (National Microbiology Data Center) accession number is NMDC20373946. The species closest in evolutionary distance to the SJ-J56 ^T^ genome is *Bacteroides kribbi*. Genome-based analysis showed that the ANI value of genomes of strain SJ-J56 ^T^ and *Bacteroides kribbi* (GCA 007341395.1) is 96.8 % and the dDDH estimation is 38.8 %. Based on these results, we concluded that the strain SJ-J56 ^T^ represents a new species of the genus *Bacteroides*, and the name *Bacteroides sphaericus* sp. nov. is proposed.

**Description of *Bacteroides sphaericus* sp. nov. (**sphae’ri.cus. L. masc. adj. *sphaericus*, spherical, referring to the cell morphology of the type strain.**)**

Cells are strictly anaerobic, spherical with single blunt end (1.3-2.1 μm long by 0.7-0.9 μm wide). Cream colored, smooth, convex colonies appear on modified 104b agar plate after 3 days of incubation. Growth occurs at range 37 °C and at pH range of 7.0-7.5. The DNA G+C content of the type strain SJ-J56 ^T^ is 42.7 mol %. The type strain SJ-J56 T (=CGMCC 1.58709^T^) was isolated from the faeces of a healthy adult.

**Taxon 2:** ***Bacteroides zhongjingi* sp. nov.**

The taxon 2, represented by strain SJ-J73 ^T^ is phylogenetically closest to *Bacteroides stercoris*, with 16S rRNA gene identity of 97.9 %. Phylogenetic tree shows that strain SJ-J73 ^T^ clusters with other members of the genus *Bacteroides* (Fig. S1), suggesting strain SJ-J73 ^T^ is a member of the genus *Bacteroides*. The genome of strain SJ-J73 ^T^ was sequenced and the NMDC (National Microbiology Data Center) accession number is NMDC20373954. The species closest in evolutionary distance to the SJ-J73 ^T^ genome is *B. stercoris*. Genome-based analysis showed that the ANI value of genomes of strain SJ-J73 ^T^ and *B. stercoris*. (GCA_000154525.1) is 97.3 % and the dDDH estimation is 30.4 %. Based on these results, we concluded that the strain SJ-J73 ^T^ represents a new species of the genus *Bacteroides*, and the name *Bacteroides zhongjingi* sp. nov. is proposed.

**Description of *Bacteroides zhongjingi* sp. nov. (**zhong.jing’i. N.L. gen. masc. n. zhongjingii, named after the physician Zhongjing Zhang, who was honored as the "Sage of Medicine" in the Eastern Han dynasty.**)**

Cells are strictly anaerobic, oval, or spherical (1.4-2.4 μm long by 0.9-1.0 μm wide). White, convex and circular colonies appear on modified 104b agar plate after 3 days of incubation. Growth occurs at range 37 °C and at pH range of 7.0-7.5. The DNA G+C content of the type strain SJ-J73 ^T^ is 43.7 mol %. The type strain SJ-J73 ^T^ (=CGMCC 1.58710^T^) was isolated from the faeces of a healthy adult.


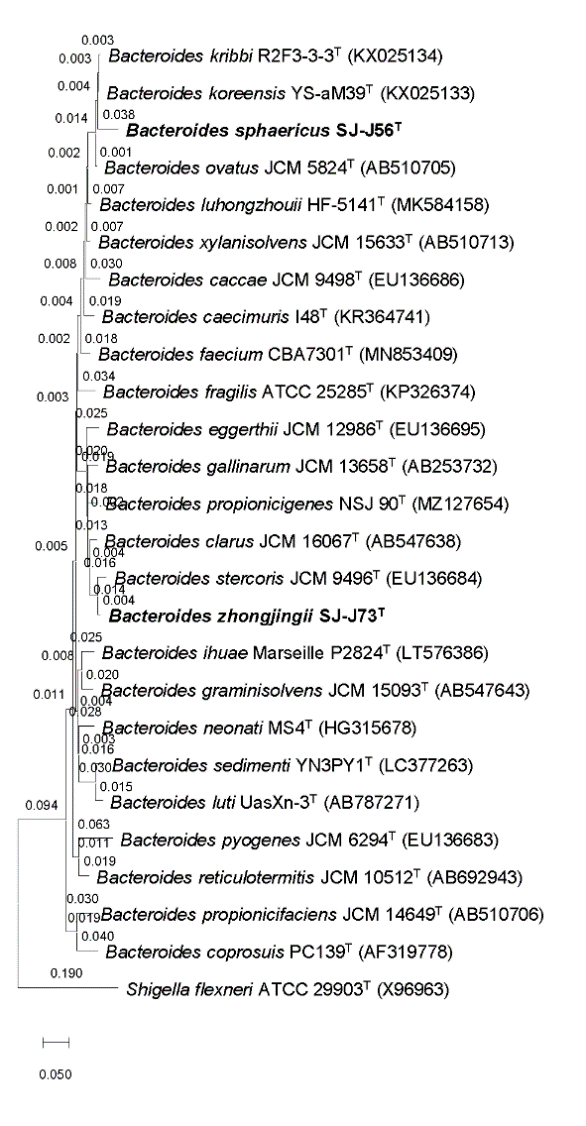


Fig. S1. Neighbour-joining phylogenetic tree based on 16S rRNA gene sequences of strains SJ-J56^T^ and SJ-J73 ^T^, together with type species of the same genus retrieved from the List of Prokaryotic names with Standing in Nomenclature. Bootstrap values were calculated from 1,000 replicates.

**Taxon 3:** ***Blautia huatuoi* sp. nov.**

The taxon 3, represented by strain SJ-J188 ^T^ is phylogenetically closest to *Blautia obeum*, with 16S rRNA gene identity of 98.1%. Phylogenetic tree shows that strain SJ-J188 ^T^ clusters with other members of the genus *Blautia,* (Fig. S2), suggesting strain SJ-J188 ^T^ is a member of the genus *Blautia*. The genome of strain SJ-J188 ^T^ was sequenced and the NMDC (National Microbiology Data Center) accession number is NMDC20373953. The species closest in evolutionary distance to the SJ-J188 ^T^ genome is *B.* *obeum*. Genome-based analysis showed that the ANI value of genomes of strain SJ-J188 ^T^ and *B. obeum*. (GCA_025147765.1) is 82.2 % and the dDDH estimation is 26.9 %. Based on these results, we concluded that the strain SJ-J188 ^T^ represents a new species of the genus *Blautia*, and the name *Blautia huatuoi* sp. nov. is proposed.

**Description of *Blautia huatuoi* sp. nov. (**hua.tuo’i. N.L. gen. masc. n. *huatuoi*, in honor of Tuo Hua, the pioneer of ancient Chinese surgical techniques and anesthesia.**)**

Cells are strictly anaerobic, spherical or oval or short rod (1.8-2.1 μm long by 1.0-1.3 μm wide). White, convex and circular colonies appear on modified 104b agar plate after 3 days of incubation. Growth occurs at range 37 °C and at pH range of 7.0-7.5. The DNA G+C content of the type strain SJ-J188 ^T^ is 43.7 mol %. The type strain SJ-J188 ^T^ (=CGMCC 1.58711^T^) was isolated from the faeces of a healthy adult.

**Taxon 4:** ***Blautia liuwansui* sp. nov.**

The taxon 4, represented by strain SJ-J190 ^T^ is phylogenetically closest to *Blautia wexlerae*, with 16S rRNA gene identity of 98.5 %. Phylogenetic tree shows that strain SJ-J190 ^T^ clusters with other members of the genus *Blautia,* (Fig. S2), suggesting strain SJ-J190 ^T^ is a member of the genus *Blautia*. The genome of strain SJ-J190 ^T^ was sequenced and the NMDC (National Microbiology Data Center) accession number is NMDC20373958. The species closest in evolutionary distance to the SJ-J190 ^T^ genome is *B. wexlerae.* Genome-based analysis showed that the ANI value of genomes of strain SJ-J190 ^T^ and *B. wexlerae*. (GCA_025148125.1) is 96.7 % and the dDDH estimation is 50.4 %. Based on these results, we concluded that the strain SJ-J190 ^T^ represents a new species of the genus *Blautia*, and the name *Blautia liuwansui* sp. nov. is proposed.

**Description of *Blautia liuwansui* sp. nov. (**liu.wan.su’i. N.L. gen. masc. n. *liuwansui*, named after the Chinese medical scientist Wansu Liu.**)**

Cells are strictly anaerobic, oval or short rod shaped (2.7-3.53 μm long by 1.2-1.4 μm wide). White, umbonate, entire margin with translucent edge colonies appear on modified 104b agar plate after 3 days of incubation. Growth occurs at range 37 °C and at pH range of 7.0-7.5. The DNA G+C content of the type strain SJ-J190 ^T^ is 41.2 mol %. The type strain SJ-J190 ^T^ (=CGMCC 1.58713^T^) was isolated from the faeces of a healthy adult.


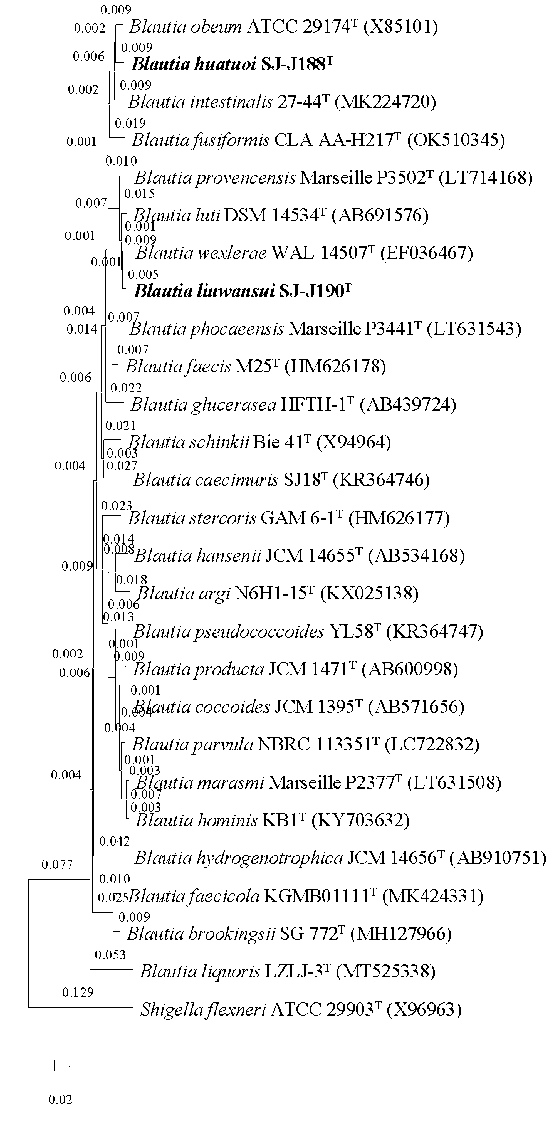


Fig. S2. Neighbour-joining phylogenetic tree based on 16S rRNA gene sequences of strains SJ-J188^T^ and SJ-J190 ^T^, together with type species of the same genus retrieved from the List of Prokaryotic names with Standing in Nomenclature. Bootstrap values were calculated from 1,000 replicates.

**Taxon 5:** ***Collinsella congzhengi* sp. nov.**

The taxon 5, represented by strain SJ-J239 ^T^ is phylogenetically closest to *Colinsela aerofaciens subsp.shenzhenensi*, with 16S rRNA gene identity of 93.8 %. Phylogenetic tree shows that strain SJ-J239 ^T^ clusters with other members of the genus *Colinsela,* (Fig. S3), suggesting strain SJ-J239 ^T^ is a member of the genus *Colinsela*. The genome of strain SJ-J239 ^T^ was sequenced and the NMDC (National Microbiology Data Center) accession number is NMDC20373948. The species closest in evolutionary distance to the SJ-J239 ^T^ genome is *Collinsella aerofacie.* Genome-based analysis showed that the ANI value of genomes of strain SJ-J239 ^T^ and *C. aerofacie*. (GCA_010509075.1) is 91.3 % and the dDDH estimation is 22.5 %. Based on these results, we concluded that the strain SJ-J239 ^T^ represents a new species of the genus *Colinsela*, and the name *Collinsella congzhengi* sp. nov. is proposed.

**Description of *Collinsella congzhengi* sp. nov. (**cong.zheng’i. N.L. gen. masc. n. *congzhengi*, named in honor of Congzheng Zhang, a prominent Chinese physician of the Jin-Yuan dynasty, recognized for his pioneering work in purgative therapy and the theory of pathogenic factors in traditional Chinese medicine.**)**

Cells are strictly anaerobic, club shaped rods with single blunt end (2.1-2.8 μm long by 0.6-1.0 μm wide). White in the center with clear margins colonies appear on modified 104b agar plate after 3 days of incubation. Growth occurs at range 37 °C and at pH range of 7.0-7.5. The DNA G+C content of the type strain SJ-J239 ^T^ is 47.9 mol %. The type strain SJ-J239 ^T^ (=CGMCC 1.58714^T^) was isolated from the faeces of a healthy adult.


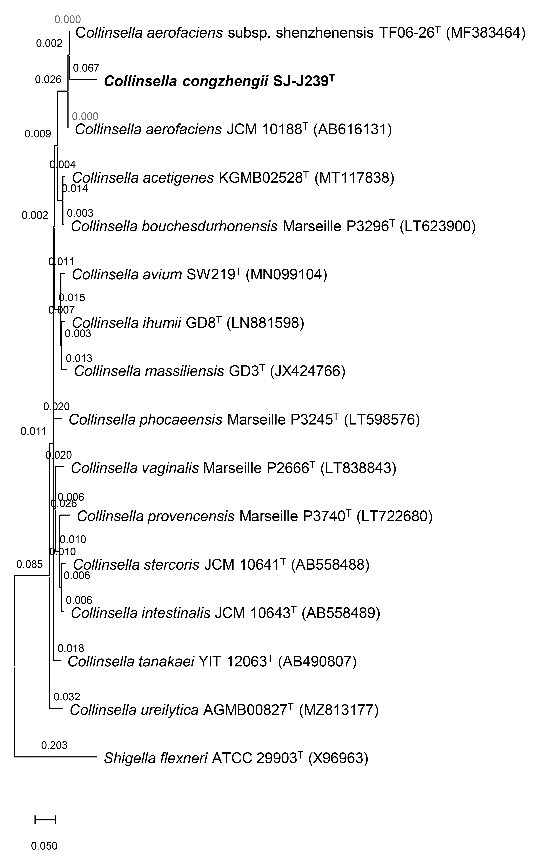


Fig. S3 Neighbour-joining phylogenetic tree based on 16S rRNA gene sequences of strains SJ-J239^T^, together with type species of the same genus retrieved from the List of Prokaryotic names with Standing in Nomenclature. Bootstrap values were calculated from 1,000 replicates.

**Taxon 6:** ***Enterococcus shizheni* sp. nov.**

The taxon 6, represented by strain SJ-J467 ^T^ is phylogenetically closest to *Enterococcus innesi*, with 16S rRNA gene identity of 93. 5 %. Phylogenetic tree shows that strain SJ-J467 ^T^ clusters with other members of the genus *Enterococcus* (Fig. S4), suggesting strain SJ-J467 ^T^ is a member of the genus *Enterococcus*. The genome of strain SJ-J467 ^T^ was sequenced and the NMDC (National Microbiology Data Center) accession number is NMDC20373950. The species closest in evolutionary distance to the SJ-J467 ^T^ genome is *E. innesi.* Genome-based analysis showed that the ANI value of genomes of strain SJ-J467 ^T^ and *E. innesi*. (GCA_022846515.1) is 94.3% and the dDDH estimation is 30.7%. Based on these results, we concluded that the strain SJ-J467 ^T^ represents a new species of the genus *Enterococcus*, and the name *Enterococcus shizheni* sp. nov. is proposed.

**Description of *Enterococcus shizheni* sp. nov. (**shi.zhen’i. N.L. gen. masc. n. *shizheni*, named in honor of Shizhen Li, whose encyclopedic Compendium of Materia Medica revolutionized pharmacognosy.**)**

Cells are strictly anaerobic, ovoid in shape, elongated along the chain direction, and appear in pairs (1.4-2.0 μm long by 0.7-0.8 μm wide). Yellow, circular, smooth, and entire colonies appear on modified 104b agar plate after 3 days of incubation. Growth occurs at range 37 °C and at pH range of 7.0-7.5. The DNA G+C content of the type strain SJ-J467 ^T^ is 50.4 mol %. The type strain SJ-J467 ^T^ (=CGMCC 1.58715^T^) was isolated from the faeces of a healthy adult.

**Taxon 7:** ***Enterococcus youxingi* sp. nov.**

The taxon 7, represented by strain SJ-J469 ^T^ is phylogenetically closest to *Enterococcus innesi*, with 16S rRNA gene identity of 94.3 %. Phylogenetic tree shows that strain SJ-J469 ^T^ clusters with other members of the genus *Enterococcus* (Fig. S4), suggesting strain SJ-J469 ^T^ is a member of the genus *Enterococcus*. The genome of strain SJ-J469 ^T^ was sequenced and the NMDC (National Microbiology Data Center) accession number is NMDC20373951.The species closest in evolutionary distance to the SJ-J469 ^T^ genome is *E. innesi.* Genome-based analysis showed that the ANI value of genomes of strain SJ-J469 ^T^ and *E. innesi*. (GCA_022846515.1) is 93.4 % and the dDDH estimation is 42.3 %. Based on these results, we concluded that the strain SJ-J469 ^T^ represents a new species of the genus *Enterococcus*, and the name *Enterococcus youxingi* sp. nov. is proposed.

**Description of *Enterococcus youxingi* sp. nov. (**you.xing’i. N.L. gen. masc. n. *youxingi*, named in honor of Youxing Wu, who pioneered the concept of epidemic pathogens as distinct entities in traditional Chinese medicine.**)**

Cells are strictly anaerobic, ovoid in shape, elongated along the chain direction, and appear in pairs (1.3-2.1 μm long by 0.7-0.9 μm wide). Small, circular, smooth colonies appear on modified 104b agar plate after 3 days of incubation. The DNA G+C content of the type strain SJ-J469 ^T^ is 41.1 mol %. The type strain SJ-J469 ^T^ (=CGMCC 1.58716^T^) was isolated from the faeces of a healthy adult.


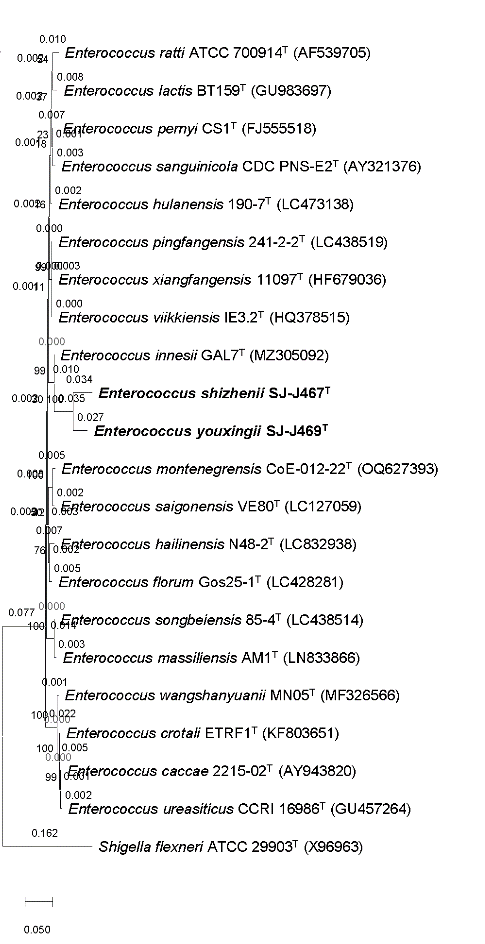


Fig. S4 Neighbour-joining phylogenetic tree based on 16S rRNA gene sequences of strains SJ-J467^T^ and SJ-J469 ^T^, together with type species of the same genus retrieved from the List of Prokaryotic names with Standing in Nomenclature. Bootstrap values were calculated from 1,000 replicates.

**Taxon 8:** ***Parabacteroides jizhoui* sp. nov.**

The taxon 8, represented by strain SJ-J690 ^T^ is phylogenetically closest to *Parabacteroides distasonis*, with 16S rRNA gene identity of 98.2 %. Phylogenetic tree shows that strain SJ-J690 ^T^ clusters with other members of the genus *Parabacteroides*, (Fig. S5), suggesting strain SJ-J690 ^T^ is a member of the genus *Parabacteroides*. The genome of strain SJ-J690 ^T^ was sequenced and the NMDC (National Microbiology Data Center) accession number is NMDC20373959. The species closest in evolutionary distance to the SJ-J690 ^T^ genome is *P. distasonis.* Genome-based analysis showed that the ANI value of genomes of strain SJ-J690 ^T^ and *P. distasonis*. (GCA_018279895.1) is 97.4 % and the dDDH estimation is 38.8 %. Based on these results, we concluded that the strain SJ-J690 ^T^ represents a new species of the genus *Parabacteroides*, and the name *Parabacteroides jizhoui* sp. nov. is proposed.

**Description of *Parabacteroides jizhoui* sp. nov. (**ji.zhou’i; N.L. gen. masc. n. *jizhoui*, named in honor of Jizhou Yang, whose codified classical acupuncture theories.**)**

Cells are strictly anaerobic, spherical with single blunt end (2.7-3.7 μm long by 1.1-1.5 μm wide). Cream colored, smooth, convex colonies appear on modified 104b agar plate after 3 days of incubation. Growth occurs at range 37 °C and at pH range of 7.0-7.5. The DNA G+C content of the type strain SJ-J690 ^T^ is 42.2 mol %. The type strain SJ-J690 ^T^ (=CGMCC 1.58721^T^) was isolated from the faeces of a healthy adult.


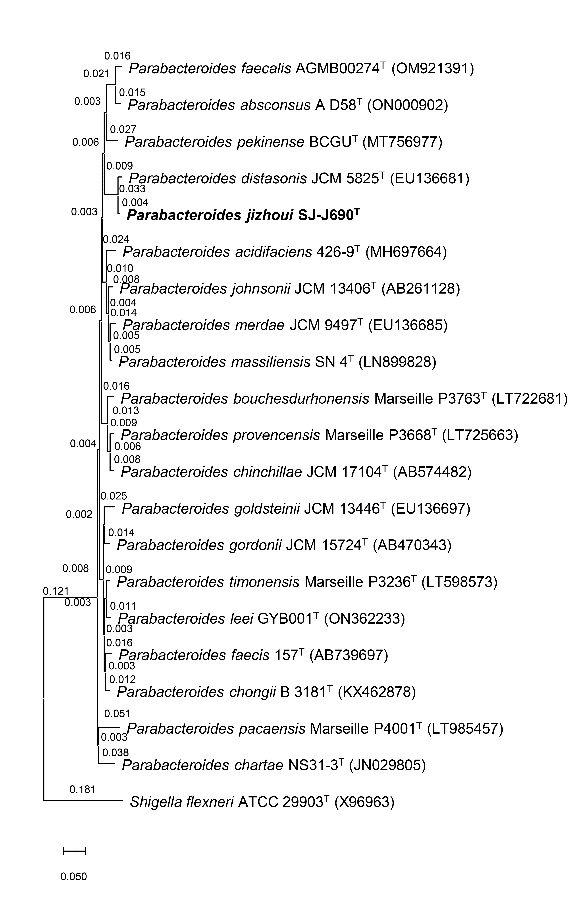


Fig. S5. Neighbour-joining phylogenetic tree based on 16S rRNA gene sequences of strains SJ-J690^T^, together with type species of the same genus retrieved from the List of Prokaryotic names with Standing in Nomenclature. Bootstrap values were calculated from 1,000 replicates.
